# Acute posterior multifocal placoid pigment epitheliopathy associated with serous retinal detachment: A case report

**DOI:** 10.1016/j.amsu.2022.103600

**Published:** 2022-04-08

**Authors:** Ahmed Mahjoub, Nadia Ben Abdesslem, Chiraz Ben Youssef, Nesrine Zaafrane, Anis Mahjoub, Atf Ben Abderrazek, Ilhem Sellem, Hanin Chtioui, Mohamed Ghorbel, Hachemi Mahjoub

**Affiliations:** a Department of Ophthalmology, Farhat Hached University Hospital, Sousse, Tunisia; b Faculty of Medicine of Sousse, University of Sousse, Tunisia

**Keywords:** Case report, Serous retinal detachment, Acute posterior multifocal placoid pigment epitheliopathy, OCT, Fluorescein angiography, APMPPE, Acute posterior multifocal placoid pigment epitheliopathy, LE, Left eye, OCT, Optical Coherence Tomography, RE, Right eye, SRD, Serous retinal detachment, VKH, Vogt-Koyanagi-Harada

## Abstract

We report the occurrence of serous retinal detachment (SRD) in acute posterior multifocal placoid pigment epitheliopathy (APMPPE).

A 22-year-old man with no general or ophthalmological pathological history presented with an acute and bilateral decreased visual acuity. There was no notion of recent flu or recent vaccination. There were anterior chamber cells and vitreous cells. Fundus revealed white-yellowish lesions, scattered on posterior pole and periphery, associated with SRD in both eyes. Fluorescein Angiography showed early hypofluorescence followed by late hyperfuorescence. Optical coherence tomography (OCT) showed hyperreflective bands of the outer nuclear layer and interruption of the ellipsoid zone associated with bilateral SRD. Repeat OCT revealed a spontaneous and complete regression of SRD in both eyes, and improvement of visual acuity after one week of evolution without any treatment.

Serous retinal detachment is an uncommon manifestation of APMPPE. It is more suggestive of Vogt-Koyanagi-Harada disease (VKH), although angiographic features are typically observed in APMPPE. Moreover, spontaneous decrease of SRD within a few days is more in favor of APMPPE.

## Highlights

• Report the occurrence of serous retinal detachment in acute posterior multifocal placoid pigment epitheliopathy.
• Enhance the difference between Vogt-Koyanagi-Harada disease and acute posterior multifocal placoid pigment epitheliopathy complicated by serous retinal detachment.
• Report the spontaneous regression of the serous retinal detachment several days after the beginning of the symptomatology without any treatment.

## 1 Introduction and importance

Acute posterior multifocal placoid pigment epitheliopathy (APMPPE) is classified as a White Dot Syndrome. It was first described by Gass in 1968 as an inflammatory chorioretinopathy [1]. It usually affects healthy and young adults and is characterized by a brutal loss of vision that occurs usually after a recent flu [2]. The fundus shows yellow-white spots corresponding to placoid lesions located in the retinal pigment epithelium. OCT and fluorescein angiography show classic features of APMPPE. Fluorescein angiography shows typically early hypofluorescent dots, followed by hyperfluorescence during the late phase. OCT shows a disruption of the inner/outer segment due to placoid lesions and hyperreflectivity of the outer retina [2,3]. Spontaneous resolution typically occurs after a few weeks of evolution. What is atypical is the presence of serous retinal detachment in APMPPE which can be confused with VKH disease [4].

This case report has been reported in line with the SCARE criteria [5].

## 2 Case presentation

**Fig. 1:**
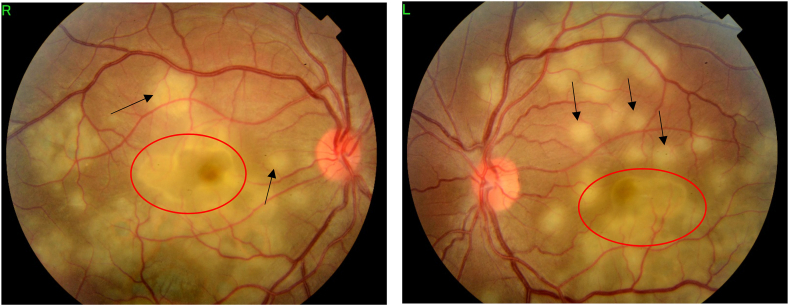
Color Fundus photograph showing multiple confluent and deep yellow-white spots involving the posterior pole (black arrows) associated with bilateral serous retinal detachment (red circles). (For interpretation of the references to colour in this figure legend, the reader is referred to the Web version of this article.)

**Fig. 2:**
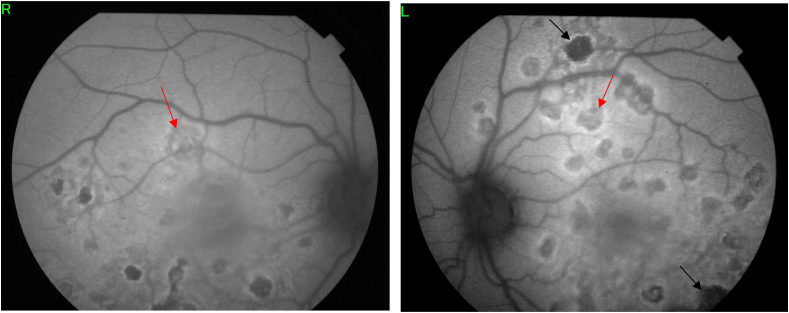
Fundus autofluorescence exhibiting hypoautofluorescent placoid lesions surrounded by hyperautofluorescent borders (Red arrows) associated to dark lesions of pigment atrophy (dark arrow). (For interpretation of the references to colour in this figure legend, the reader is referred to the Web version of this article.)

**Fig. 3:**
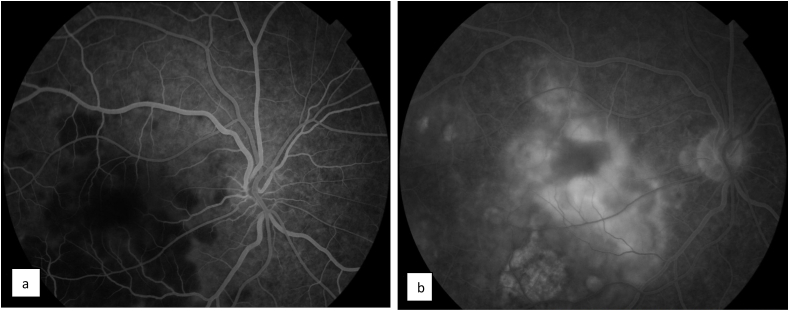
Fluorescein angiography of the right eye revealing early hypofluorescence (a) followed by late hyperfluorescence of placoid lesions (b).

**Fig. 4:**
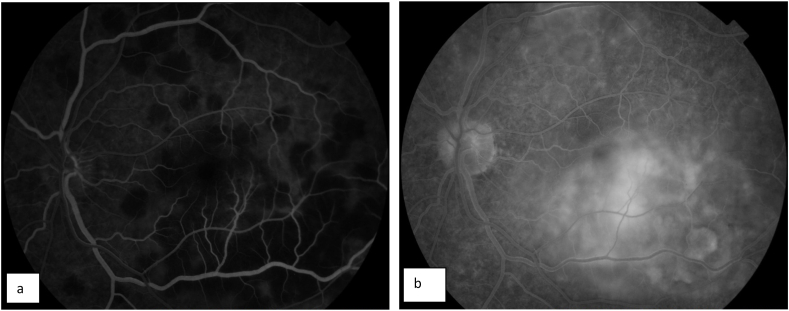
Fluorescein angiography of the left eye showing early hypofluorescence (a) followed by late hyperfluorescence of placoid lesions (b).

**Fig. 5:**
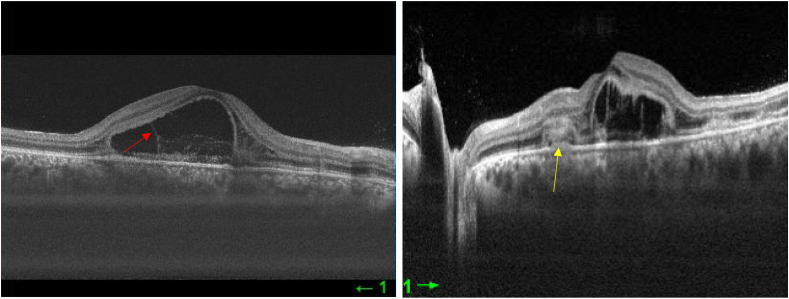
Macular OCT showing bilateral serous retinal detachment with multiple septae (red arrow) associated to placoid lesions in the outer layer retina and interruption of the ellipsoid zone (yellow arrow). (For interpretation of the references to colour in this figure legend, the reader is referred to the Web version of this article.)

A 22-year-old Caucasian man with no family history, no drug/psychosocial history and no general or ophthalmological pathological history who presented to the emergency department with an acute and bilateral decreased visual acuity more marked on the right eye. There was no notion of recent flu or recent vaccination. On examination, best corrected visual acuity was limited to counting fingers at 2 m in the right eye (RE) and was 2/100 in the left eye (LE). The cornea was clear. Examination of the anterior segment revealed 2+ of anterior chamber cells in both eyes. Intraocular pressure was normal. Examination of the posterior segment showed 2+ vitreous cells bilaterally. Fundus examination showed multiple, deep, white-yellowish lesions with blurred borders, scattered throughout the posterior pole and periphery, associated with SRD in both eyes (Fig. 1). Autofluorescence images showed hypoautofluorescent lesions surrounded by hyperautofluorescent borders and dark lesions of pigmentary atrophy (Fig. 2). Fluorescein angiography showed early hypofluorescence followed by hyperfluorescence during the late phase and there was no vasculitis (Fig. 3, Fig. 4). Macular OCT revealed bilateral SRD with multiple septa associated with hyperreflective bands of the outer nuclear layer and interruption of the ellipsoid zone (Fig. 5).

**Fig. 6:**
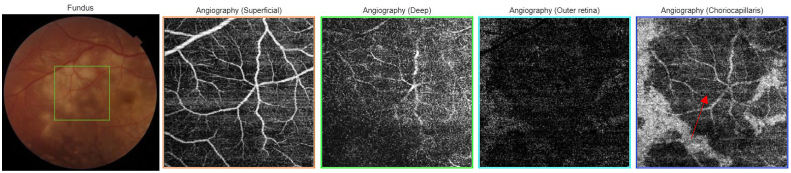
OCT Angiography of the right eye showing dark spots located in the choriocapillaris corresponding to placoid lesions with vascular rarefaction in the choriocapillaris (red arrow). (For interpretation of the references to colour in this figure legend, the reader is referred to the Web version of this article.)

**Fig. 7:**
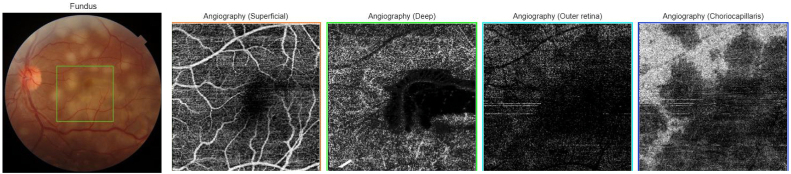
OCT Angiography of the left eye showing dark spots located in the choriocapillaris corresponding to placoid lesions with vascular rarefaction in the choriocapillaris. (For interpretation of the references to colour in this figure legend, the reader is referred to the Web version of this article.)

OCT Angiography showed dark spots located in the choriocapillaris (Fig. 6, Fig. 7).

Syphilis serology and Mantoux test were negative, chest X ray was normal, angiotensin-converting enzyme and blood calcium level were normal. Neurological examination showed no signs of neurological involvement that may raise the suspicion of cerebral vasculitis associated with APMPPE.

In front of the aspect of the SRD compartmentalized in septa we discussed the diagnosis of VKH but the evolution without any treatment and the absence of general symptoms were against this diagnosis.

We also evoked tuberculosis which may be represented by multifocal choroiditis, but Mantoux test was negative and chest X ray was normal. Before starting any eventual corticosteroid medication, the diagnosis of tuberculosis and syphilis was eliminated.

Sarcoidosis is also a differential diagnosis of APMPPE, but clinical presentation was not in favor. Moreover angiotensin-converting enzyme and blood calcium level were normal.

Multiple white dot syndromes were discussed such as idiopathic multifocal choroiditis, birdshot chorioretinopathy, punctate inner choroidopathy, but were easily eliminated by clinical characteristics.

**Fig. 8:**
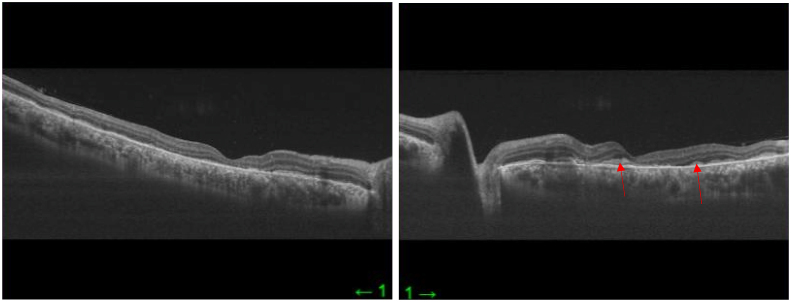
Repeat macular OCT showing complete regression of the serous retinal detachment after one week of evolution. A few placoid lesions remain in the outer layer retina in the left eye (red arrows).

The diagnosis of APMPPE was made. After one week of evolution, repeat OCT (Fig. 8) showed a spontaneous and complete regression of SRD in both eyes, and improvement of visual acuity. Best corrected visual acuity after one week was 20/50 in the RE and 20/100 in the LE, without any treatment. The patient received topical steroid drops during hospitalization to reduce local inflammation and was discharged after ten days without any treatment due to spontaneous improvement of the symptoms. A follow-up examination, performed after one month of discharge, revealed a significant improvement in symptoms. Best corrected visual acuity was 20/20 in both eyes and local inflammation totally disappeared.

This case report has been reported in line with the SCARE criteria [5].

## 3 Clinical discussion

APMPPE is characterized by inflammatory lesions at the outer retina and choriocapillaris. It is usually associated to minimal inflammation of the anterior chamber as well as a vitritis. However, serous retinal detachment is an uncommon complication [4]. Dome-shaped detachment with multiple septa is rather in favor of Vogt-Koyanagi-Harada (VKH) disease [3] but can also be seen in APMPPE as was noted in this case.

The initial description of APMPPE by Gass in 1968 did not include the presence of SRD in this condition [3]. A few cases of SRD have been found in APMPPE, and differential diagnosis with VKH disease was sometimes difficult. However, typical appearance on fluorescein angiography with multiple dark spots corresponding to placoid lesions with early hypofluorescence followed by late and pronounced placoid hyperfluorescence, absent pinpoint leakages, minimal inflammation, absence of general symptoms, and often spontaneously favorable evolution after a few months allow to correct the diagnosis of APMPPE [6]. In addition to SRD, the lesions on OCT are represented by hyperreflectivity of the outer retinal layers, with interruption of the ellipsoidal zone by placoid lesions, suggestive of APMPPE. OCT Angiography shows dark spots corresponding to placoid lesions. These dark spots are rather hypoperfusion of the choriocapillaris than blockage of the flow signal by retina outer layer [7].

It seems that the pathophysiology of SDR in APMPPE begins at the level of choriocapillaris. The choriocapillaris ischemia seen in APMPPE causes increased vascular permeability that induces exudative retinal detachment. Ischemia will secondary affect outer retinal layers [8] with interruption of the pigment epithelium and appearance of placoid lesions.

Spontaneous regression of SRD without any corticosteroids is not in favor of VKH disease, which is very responsive to corticosteroids, and reinforces the diagnosis of APMPPE. According to Goldenberg and al, APMPPE is classified into four stages, including an early stage with serous retinal detachment and later stage with complete disappearance of SRD [9].

Rapid resolution of subretinal fluid can explain why SRD is rarely seen in cases of APMPPE. It usually appears in the acute phase and lasts only a few days.

Our case report showed that SRD is an uncommon complication, that it can spontaneously regress without any treatment, and is not a prognostic factor for poor visual outcome. Quick resolution can explain why SRD is not a frequent sign in APMPPE.

In this case we could not prove the action of corticosteroids on SRD due to quick resolution. However some authors indicate corticosteroids specially when SRD is associated to papillitis [6].

According to Kitamura and al, indication of treatment in APMPPE is not quite established. Resolution of SRD suggest that there is a spontaneous cessation of choroidal inflammation [10]. Therefore, SRD is not an absolute indication for corticosteroid treatment in APMPPE.

## 4 Conclusion

Serous retinal detachment is an acute complication of APMPPE, and it completely disappears after few days of evolution and most of the time without any treatments. SRD with lobulated septa can lend to confusion with VKH disease but clinical manifestations, angiography and especially evolution redress the diagnosis of APMPPE.

## Funding

No funding or grant support.

## Authorship

All authors attest that they meet the current ICMJE criteria for.

## Provenance and peer review

Not commissioned, externally peer reviewed.

## Patient consent

Written informed consent was obtained from the patients for publication of this case report and accompanying images. A copy of the written consent is available for review by the Editor-in-Chief of this journal.

## Guarantor

Atf ben abderrazek, resident.

## Provenance and peer review

Not commissioned, externally peer reviewed.

## Ethical statement

We further confirm that any aspect of the work covered in this manuscript that has involved human patients has been conducted with the ethical approval of all relevant bodies and that such approvals are acknowledged within the manuscript. IRB approval was obtained (required for studies and series of 3 or more cases) Written consent to publish potentially identifying information, such as details or the case and photographs, was obtained from the patient(s) or their legal guardian(s).

## Author contribution

Ahmed Mahjoub: writing the paper. Nadia Ben Abdesslem: data analysis. Chiraz Ben Youssef: writing the paper. Nesrine Zaafrane: data collecting. Anis Mahjoub: study concept. Atf Ben Abderrazek: study design. Ilhem Sellem: data interpretation. Hanin Chtioui: data interpretation. Mohamed Ghorbel: correcting the final paper. Hachemi Mahjoub: correcting the final paper.

## Declaration of competing interest

The following authors have no financial disclosures: (Ahmed Mahjoub, Nadia Ben Abdessalem, Chiraz Ben Youssef, Nesrine Zaafrane, Anis Mahjoub, Atf Ben Abderrazek, Ilhem Sellem, Hanin Chtioui, Mohamed Ghorbel, Hachemi Mahjoub).
